# Using T-Cell Subsets to Better Characterize Immunoresiliency and Immunodeficiency in Patients with Recurrent Infections

**DOI:** 10.3390/idr16060097

**Published:** 2024-12-16

**Authors:** Justine Hung, Bryan Vonasek, Daniel Rosenberg, Tri Vo, Rob Striker

**Affiliations:** 1 Department of Medicine, University of Wisconsin-Madison, Madison, WI 53706, USA; 2 Department of Biochemistry, University of Wisconsin-Madison, Madison, WI 53706, USA; 3 Department of Pediatrics, Michigan State University, East Lansing, MI 48842, USA

**Keywords:** CD4/CD8 ratio, immunodeficiency, common variable immunodeficiency

## Abstract

Background/Objectives: Common Variable Immunodeficiency Disease (CVID) and other immunodeficiencies can present in subtle and variable ways. Whether or not a genetic lesion can be identified, there are not well understood biomarkers that quantitatively describe how severe a deficiency is. Here we discuss two possible ranking systems, CD4/CD8 T cell ratios and Immune Health Grades, and how such data maybe applicable to some immunodeficiencies. Methods: This is not a systematic review, but we identify papers relating to immunodeficiencies with enough data to comment on the CD4/CD8 and Immune Health Grade. We also summarized relevant data publicly available from USIDNET, a website that compiles data on immunodeficiencies, and provide two new cases that illustrate ways that this information can alter clinical assessment. Results: We review the HIV literature on CD4/CD8 T cell data and how this correlates with both immunologic function and comorbidity better than CD4 count alone. The ratio aslso relates to a new system called Immune Health Grades (IHG) derived from young adult to elderly subjects from many NIH cohorts without HIV. CVID is often thought of as an antibody problem, but in fact most patients also have low CD4/CD8 ratio and other cellular abnormalities. We review IDNET to categorize nine molecular immunodeficiencies including two subcategories of CVID into low, normal, or high ratios. Finally, we present two new cases in the literature of patients with recurrent infection and discuss how viewing the cases through the “lens” of CD4/CD8 ratio and IHG can facilitate clinical decisions. Conclusions: Emerging data suggests at least some immunodeficiencies can be grouped by how abnormal their CD4/CD8 ratio or IHG. This represents a clinically available biomarker that can be tracked to see if the condition is worsening or not.

## 1. Introduction

Immunodeficiencies are more commonly diagnosed during childhood; however, certain immunodeficiencies such as common variable immunodeficiency (CVID) may only become apparent during adulthood [1]. Sometimes it is impossible to know if an immunodeficiency is a “primary inborn error of immunity” or something that developed due to an exposure, or a combination of multiple subtle variants/mutations in genes or epigenetics. Regardless of age and cause, immunodeficiency should be considered in individuals presenting with recurrent infections and/or opportunistic infections. While time is needed to distinguish between bad luck versus a true, persistent immunodeficient state, categorizing that persistent state into a diagnosis with a known natural history is often challenging. Recently, the concept of immune resilience has been introduced and involves T-cell flow cytometry results to define four categories of immune health grades (IHGs) ranging from IHG-I to IHG-IV (Table 1) [2,3]. The IHG metric uses the CD4/CD8 ratio with a ratio >1.0 (IHG-I and II) correlating with optimal immune resiliency, while a ratio <1.0 is defined as a “CD4-CD8 disequilibrium” (IHG-III and IV) state and is associated with a lower ability to recover from new burdens from various antigens [2]. IHG-I has an absolute CD4 count of >800 cells/mL and is associated with optimal immune resiliency, evidenced by resistance to severe COVID-19 infection, severe influenza, sepsis, and acquiring HIV [3]. In contrast, lower grades have progressively worse outcomes. Extensive modeling was used to divide what is likely a continuum into four grades, with grades 2–4 showing evidence of elevated cytokines, and immune exhaustion. This was based on data from fifteen human NIH cohorts, totaling 50,000 subjects, and corroborated by controlled experiments in nonhuman primates and mice [3]. The majority of the population at most ages is IHG-I; however, the proportion of IHG-I declines progressively after age 30. Only about 25% of the population over the age of 90 are IHG-I. There is an estimated 5% of the population under the age of 30 that are IHG-III or IV [3]. In this review, we go over several uncommon human genetic variants that can present as CVID or other immunodeficiency syndromes. We describe the importance of characterizing their T-cell subsets in addition to their associated low antibody levels that define CVID. We also provide some real-world examples of T-cell subset assessments in immunodeficient patients. We suggest that the CD4/CD8 cell ratio (or IHGs) can be utilized to gain insight into anticipated complications related to a patient’s immunodeficiency by using more well-understood monogenetic diseases with similar CD4/CD8 cell ratios as a guide. For this reason, we propose that further research into how CD4/CD8 ratios may correlate to the disease course of people with suspected CVID is a worthwhile area of focus.

Cutoffs vs. continuums: Flow cytometry equipment is now standard clinical lab equipment at most hospitals in the developed world. It also exists in many reference hospitals in developing nations. Accurate counts do require enumerating total lymphocytes, and, typically, cells are not frozen prior to counting. The two largest lymphocyte populations are CD4 T cells and then CD8 T cells in most patients, both of which are typically more numerous than B lymphocytes. The IHG system incorporates both the CD4/CD8 T-cell ratio as well as the CD4 count to delineate between IHG categories. While the concept of IHGs is an important advance, it essentially considers a patient with a ratio of 0.9 and a CD4 count of 700 cells/mL (IHG-IV) as more immunocompromised than a patient with a ratio of 0.2 and a CD4 cell count of 850 cells/mL (IHG-III). This grouping of a relatively “high” CD4 T-cell count overriding a low CD4/CD8 T-cell ratio contradicts the literature of treated patients with HIV [4,5,6]. It remains unclear if a ratio of 1.5 corresponds to a “better” immune system than a ratio of 1.1, but as the ratio falls below 1.0, immune incompetence becomes increasingly obvious. There is now more data supporting the concept that patients with low CD4 count counts but without HIV (some of whom also have low ratios) are vulnerable to specific and severe infections as well as certain cancers [7]. There is emerging literature on IHGs that suggests that the optimal IHG protects against both the acquisition of HIV as well as the ability to control the HIV viral load independent of medical treatments [3]. Even relatively small studies have demonstrated a relationship between CD4/CD8 T-cell ratios at or below 0.3 with increased infection and cancer risk [8,9]. On the other hand, the IHG system also considers all ratios >1.0 as “optimal” even though evidence suggests that ratios greater than 5 may actually represent more severe physical impairment in elderly people who are CMV-positive [10] and smoking can also increase the ratio above “normal levels” [11]. There is likely an optimal ratio that is above one but not “too high.”

The clinical significance of CD4 cell counts between 400 and 800 cells/mL remains debated, and the amount of time a person lives with a low CD4 cell count likely plays a role in clinical significance [12]. Notably, given the extremely low lymphocyte counts being incorporated into these analyses, variations between lab interpretations across facilities must also be considered. Broadly, both a CD4/CD8 cell ratio below 1.0 and a CD4 cell count below 800 cells/mL likely indicate a degree of immune incompetence/lack of resilience. This information may provide useful insight into projected disease courses of individuals undergoing an immunodeficiency workup. The specifics on how these two factors can be incorporated to differentiate between different levels of impaired immune function need continued investigation. Small deviations are likely more significant if they persist over years. However, there is literature showing nonopportunistic infections occurring more frequently in people with HIV with a CD4 count above 200 cells/mL [12], and, biologically, there is no clear evidence of a threshold effect until the CD4 count is above 800 cells/mL. Finally, development of the IHG system has largely relied upon data from adult studies. Although IHG principles likely also apply to the pediatric population, given that T-cell maturation is highly dynamic early in life and that normal ranges of CD4 and CD8 counts vary from infancy into adulthood, more research is needed to evaluate how well the IHG ratio cutoff of 1.0 and CD4 count cutoff of 800 cells/mL apply to the pediatric population.

Common Variable Immunodeficiency (CVID): An independent but overlapping label of a persistent immunodeficient state is CVID, which is a heterogeneous syndrome that can be difficult to recognize in specific patients. The fundamental feature of CVID as described by Charles Janeway Sr. in 1953 is low antibody levels [13]. Missing in the description of CVID is that approximately half of CVID patients also have demonstrable T-cell defects [14,15]. CVID is present in ~1 in 25,000–50,000 people worldwide [13,14]. Individuals with CVID are treated with immunoglobin (Ig) replacement therapy. In an assessment of 473 CVID patients receiving Ig replacement therapy, a significant improvement in overall mortality was found compared to patients with CVID assessed prior to the establishment of Ig replacement as a standard therapy. However, both male and female subjects still had higher overall mortality compared to sex- and age-matched controls [16]. There is no one genetic or environmental cause of CVID, and CVID often coexists with autoimmune, immunoproliferative, or other diseases. CVID is likely polygenic [1], but whether it is most commonly a genetic disease is up for debate. Epigenetic methylation may also play a role in CVID as B cells of CVID patients have been shown to have an impaired ability to demethylate and up-regulate genes involved in transitioning from naïve to memory B cells [17].

The diagnosis of primary immunodeficiencies including CVID remains challenging with delayed diagnosis affecting quality of life being an ongoing issue [18]. A diagnosis of CVID is typically made after the age of four. In order to make a diagnosis of CVID, there should be a demonstration of hypogammaglobulinemia in general, and the following characteristics should be met:

(1) Significantly reduced total serum concentrations of IgG;
(2) Low IgA and/or IgM;
(3) Poor or absent response to vaccination;
(4) The exclusion of other immunodeficiency states (protein losing states, HIV, immunosuppressant drugs, etc.).

None of the above criteria involve low T cells or altered T-cell subsets. Nevertheless, about 80% of the sixty CVID patients with additional controls evaluated by Giovanetti et. al. had either a low naïve CD4 cell count, a high CD8 cell count, or both relative to healthy controls. Furthermore, in that study, the degree of T-cell abnormality correlated with clinical symptomatology [14]. In an even larger study of 248 CVID patients, although CD4 and CD8 T-cell numbers were not directly reported, they did report that 40% of subjects had abnormally low T-cell proliferation to one or more antigens [15]. These studies suggest that T-cell subsets, including a low CD4/CD8 ratio (or IHG II-IV), which have already been associated with poor immune resiliency, may be an important datapoint in characterizing CVID. Unfortunately, while we know low CD4/CD8 ratios are common in CVID, we do not have longitudinal data. There are likely multiple reasons that CVID is not a well-defined disease as several different combinations of genetics and exposures could lead to low immunoglobulin levels. Additionally, while CVID is typically diagnosed in a setting of recurrent infections, noninfectious complications, such as autoimmune disease, lymphoproliferative disease, and malignancy, have also been linked with CVID [1]. Patients can have defects in one or multiple small, T-cell populations, such as NK cells, NKT cells, gamma/delta T-cells, or other potentially important lymphocyte populations. Yet enumerating these many small populations and giving reliable reference values is challenging for many clinical labs. CD4/CD8 ratios, however, are accessible pieces of data that can be obtained through routine flow cytometry. Given the fact that Ig therapy can improve overall mortality in people with CVID [15,18] but is a significant time commitment for both patients and providers, continuing to refine the diagnostic process is important for identifying which individuals are at greatest risk for significant infections and would benefit from the therapy the most.

Genetics and Primary Immunodeficiencies: Over 400 different defective or absent genes have now been identified as causes of primary immunodeficiency [19]. When faced with a patient who has recurrent infections, a clinician does not know if the problem is “primary” or acquired. Labs such as T-cell subsets can help with conducting a risk assessment regardless of whether the problem is primary or acquired. Antibody deficiencies are the most common type of primary immunodeficiency [20] A useful resource is the U.S. Immunodeficiency Network (USIDNET), which currently provides data on over 5000 patients with a primary immunodeficiency. About half of the patients have chromosomal and monogenetic lesions that explain their immunodeficiency [21]. Additionally, different mutations within the same gene can have varying effects, and not enough examples have been studied to know if there is a consistent effect between individual genetic defects and the CD4/CD8 ratio. For example, while a variant in PIK3 might raise the CD4/CD8 T-cell ratio, not all functionally significant variants will necessarily have the same effect [22]. Nevertheless, some genetic conditions do alter the CD4/CD8 ratio in a consistent way. Traditionally, the ratio has not been used in distinguishing between different immunodeficiencies. Many immunodeficiencies lack enough data to fully assess their relationship with the CD4/CD8 ratio due to small patient populations. Much of our “norms” from ratios come from adult data, whereas some immunodeficiencies are primarily from the diagnosis of children, so more data are needed to improve comparisons. In Table 2 and Table 3, we have complied a few of the more common immunodeficiencies such as Wiskott–Aldrich Syndrome (252 cases on USIDNET) and outlined their associated symptoms and CD4/CD8 ratios and IHGs. IHGs were not assigned to ratios over two given the unclear clinical significance associated with high CD4/CD8 ratios [10].

Less common immunodeficiencies are described as well with their respective CD4/CD8 ratios. A range of CD4/CD8 ratios are represented; however, small patient populations are being represented for some of the less common immunodeficiencies.

Table 2 lists some immunodeficiency diagnoses that can alter both T cells and B cells as well as antibody levels where there is at least some data on the CD4/CD8 ratio that can be found for the patients. Table 3 lists diagnoses in which there are no known defects in B cells/antibody levels. Additional information is needed for these and other immunodeficiency syndromes to fully assess if there is a correlation between the CD4/CD8 ratio and disease severity.

### Examples of Genetic Conditions Presenting as CVID

Inducible co-stimulator (ICOS) deficiency: Inducible co-stimulator (ICOS) deficiency was first linked to CVID in 2003 [35]. In the years following, more than 15 patients have been identified with this deficiency and have had long-term follow-up. A total of 3 of these 15 patients had persistently inverted ratios (see Table 2) [27]. ICOS plays a role in the signaling cascade that induces proliferation and cell survival in T cells. It is also important in the maintenance of germinal centers in the lymphatic system and is, therefore, important in the formation of memory B cells. Deficiency in ICOS has been linked with increased susceptibility to viral infections, opportunistic infections, and certain cancers. Most patients have been found to have impaired B-cell development as well as decreased CD4+ T-helper cells [27,28]. This implies that ICOS deficiency may be a combined B- and T- cell immunodeficiency as opposed to solely being a B-cell deficiency. Treatment for ICOS deficiency typically involves immunoglobulin substitution and treatment of infections that arise. In a second paper with ICOS deficiency, three of nine subjects had ratios <1.0 and two of these patients with a CD4/CD8 ratio <1.0 were from the same family. A family member with ICOS deficiency but a CD4/CD8 ratio >1.0 had remained asymptomatic while their dizygotic twin with a CD4/CD8 ratio <1.0 had already met diagnostic criteria for CVID and has been receiving Ig therapy [27].

Cytotoxic T-lymphocyte-antigen-4 (CTLA-4) deficiency: CTLA-4 is a necessary component for regulatory T-cell function and serves as a negative immune regulator. Variants in CTLA-4 have been associated with autoimmune conditions as well immune deficiencies. In a large retrospective cohort study of CTLA-4 variant carriers worldwide, 26% had a diagnosis of CVID. Clinical manifestations of CTLA-4 deficiency varied, though some of the most common manifestations included hypogammaglobulinemia (84%) and lymphoproliferation (73%) [29]. Recurrent respiratory system infection was a common manifestation, with one patient with CVID requiring a lung transplant due to parenchymal lung damage that had occurred in a setting of recurrent infection. Diarrhea was also seen in over half of the cohort with the inciting pathogen rarely identified [27]. Overall, those with CTLA-4 had a wide range of physical manifestations with an estimated clinical penetrance among variant carriers of 67% based on this cohort. While the majority of CD4/CD8 cell ratios for affected patients carrying a CTLA-4 variant were above one, there was notably a greater reduction in absolute CD4+ T cells between affected and unaffected individuals with the CTLA-4 variant compared to the reduction seen in absolute CD8+ T cells between the two groups [29].

New clinical cases not previously reported as examples of how ratio/IHG information may provide clinical context: The first case is a 64-year-old female with a 10+ year history of a multi-system lymphomatoid granulomatous disease. On initial presentation, she was found to have leukopenia and neutropenia with a bone marrow biopsy showing multiple granulomas, suggesting a diagnosis of sarcoidosis. An inguinal lymph node biopsy also suggested sarcoidosis. She was initially treated with prednisone; however, methotrexate was added due to an insufficient response. Despite the addition of methotrexate, lymphadenopathy worsened. A core lymph node biopsy was positive for a lymphoproliferative disorder. She was initially treated with rituximab at the time of diagnosis with a partial response on imaging, and she was subsequently treated with rituximab, cyclophosphamide, doxorubicin hydrochloride, vincristine, and prednisone (R-CHOP). She underwent an immunodeficiency workup and was found to have low immunoglobulin levels (IgG 446, IgA 97, and IgM 34 mg/dL) though not low enough to meet diagnostic criteria for CVID. Yet, with low immunoglobulin levels and persistent lymphadenopathy, she was started on intravenous immune globulin (IVIG) for about 6 months. Her condition later improved and she was continued on chronic low-dose prednisone with relative stability for about 6–7 years. In 2022, she was hospitalized for disseminated varicella infection. During this hospitalization, immunology was consulted for an immunodeficiency workup that was significant for findings of hypogammaglobulinemia (IgG 426, IgA 46, and IgM 7 mg/dL) and B- and T-cell lymphopenia. Notably, the absolute CD4 count was 36 cells/uL, and the CD8 count was 1125 cells/uL for a CD4/CD8 T cell ratio <0.01 (IHG IV) despite only a distant history of chemotherapy. IVIG was continued and genetic testing was initiated to assess for an underlying immunodeficiency. Genetic analysis thus far has found a pathogenic genetic variant in FANCL and a variant of uncertain significance in ZCCHC8 and SAMD9. She was also found to have critically short telomeres. Per discussions between immunology and hematology, her presentation did not appear to be consistent with diseases associated with the above genes (Fanconi anemia, SAMD9/MIRAGE syndrome, and telomere biology disorder). A full genome analysis was declined by the patient. Even though a molecular or even syndromic diagnosis remains elusive for this patient, the workup has been revealing as to the severity of her condition. The value of obtaining lymphocyte subsets in this case is the finding that both her CD4 count and CD4/CD8 ratio/IHG are very abnormal, which validates her symptoms and the measures taken to treat them as well as providing some context for the severity of her varicella infection.

The second case is a 45-year-old male with a history of recurrent sinus infections, splenomegaly, and recurrent fevers of unknown origin starting in his late 20s who was diagnosed with CVID in his early 30s. Notably, he had an extensive autoimmune family history of lupus, Hashimoto’s disease, and Addison’s disease in his immediate family, though no known primary immunodeficiencies. He initially met the criteria for a CVID diagnosis, yet he declined IVIG therapy given that his symptoms were relatively tolerable, and he had not experienced severe infection requiring hospitalization for several years. He had previously undergone an extensive workup a few years prior to assess for a possible primary immunodeficiency. The initial workup was significant for IgG 318 mg/dL, IgA < 5 mg/dL, IgM 11 mg/dL, absolute CD4 count of 143 cells/uL, and absolute CD8 count of 219 cells/uL with a CD4/CD8 T-cell ratio of 0.66 (IHG of IV). Genetic testing was consistent with a diagnosis of autosomal dominant familial Bechet-like autoinflammatory syndrome. The genetic variant found led to a nonsense mutation in the gene encoding TNFAIP3, which is a zinc finger protein and ubiquitin-editing enzyme known to inhibit nuclear factor kappa B (NF-kB) activation and tumor necrosis factor-mediated apoptosis [36]. The TNFAIP3 reading frame was truncated at codon 183, leading to a high suspicion that he had a pathogenic variant. He was subsequently referred to rheumatology for co-management. Symptoms included ongoing sinus infections, occasional rash and pruritis in the extremities, and intermittent low-grade fever and night sweats. Currently, he is being treated with colchicine twice daily for symptoms, with consideration of anti-IL-1b therapy as the next step. The patient recently started IVIG treatments for recurrent infections and hypogammaglobulinemia. This is another case of low antibody levels in the setting of an abnormal ratio and IHG. Acknowledging this latter factor can be helpful for encouraging patients to accept some therapeutic options.

## 2. Discussion

The causes (both genetic and nongenetic) of immunodeficiency are complex and difficult to pin down. It has been recognized that CVID often includes individuals with T-cell problems [14,15]. However, CVID remains to be viewed primarily through a “B cell lens” without quantitation of T-cell alterations or subsets. While T-cell subsets are occasionally incorporated into the diagnostic workup of those with CVID, what to do with the information is not widely understood. How to separate CVID from other immunodeficiencies that alter both humoral and cellular immunodeficiency is not clear as well. Occasionally, genome sequencing can pinpoint a genetic lesion, which can help with assessing prognosis. However, the majority of the time, genome sequencing does not provide a simple answer as to why a particular patient is immunodeficient [7]. Even a relatively simple physiologic description such as “idiopathic CD4 cytopenia” can be divided into low, medium, and high CD8 counts. In an analysis of individuals with idiopathic CD4 cytopenia, those with a CD8 count in the middle third had a lower risk for opportunistic infection [7]. Many diseases that have an “immune component” may skew the CD4/CD8 ratio such as multiple sclerosis [37] and emphysema [38]. Although in the case of smoking the kinetics of when smoking [39] stops raising the ratio and when the ratio starts to fall remain quite unclear.

## 3. Conclusions

Overall, the process of establishing a diagnosis to explain an immunodeficient state and predicting disease severity based on the diagnostic process remains challenging. The CD4 and CD8 T-cell count as well as the ratio between the two with the IHG represent an additional consideration that can be applied to the diagnostic process for primary immunodeficiencies including CVID, which has been historically assessed with a focus on B-cell function. In CVID particularly, it can be difficult to predict how much regular IVIG infusions will change the natural history of a specific patient, so biomarkers can be helpful. As shown in the emerging IHG classification system, as well as the cases and genetic conditions described above, a lower CD4/CD8 T cell ratio and IHG classification III and IV can correlate with a worse prognosis. The CD4 and CD8 T-cell count is a more accessible data point that can serve as a possible predictor of symptoms and outcomes of people awaiting a specific diagnosis, and is a worthwhile area for more studies to elucidate the clinical significance.

## 4. Future Directions

While we have reviewed case reports and large datasets justifying the utility of measuring ratios, clearly more data are needed. The implications of higher ratios (over three) are particularly glaring. The ongoing issue of delayed diagnosis of immunodeficiencies calls for a more robust diagnostic process. Genetic testing remains an emerging component of this process, but the large majority of people with immunodeficiencies likely have more complex conditions than cannot be revealed by genome sequencing. Suggestions for potential areas of study could come in the form of a retrospective analysis of infection risk in patients with CVID with varying CD4/CD8 ratios as well as analyzing for any potential mortality effects related to CD4/CD8 ratios. There is a particular need for examining IHGs in pediatric populations. Whether or not the ratio or IHGs become a common, clinically useful tool outside of HIV remains to be seen.

## Figures and Tables

**Table 1:** Immune health grade classification system [2].

IHG Class	CD4/CD8 Ratio	CD4+ Level
I	≥1.0	≥800
II	≥1.0	<800
III	<1.0	≥800
IV	<1.0	<800

**Table 2:** Immunodeficiencies that include both T-cell and humoral defects.

Disease	CD4/CD8 Ratio	Estimated IHG Class	Genetic Defect and Inheritance	Associated Features	Reference
1a. CD4/CD8 < 1.0					
X-linked Bruton Type Agammaglobulinemia	Ratio < 1 from age 8 to 14 N = 10	III/IV	Bruton’s kinase X-linked	Slow growth, immunodeficiency	[23]
MHC Class II Deficiency	Ratio < 1 N = not reported	III/IV	CIITA, RFX5, RFXAP, FRXANK Autosomal recessive	Recurrent, severe infection, GI infection, failure to thrive	[24]
Adenosine Deaminase Deficiency (those presenting as SCID)	Ratio 0.39 (range 0.1–7.0) N = 7	II-IV	ADA Autosomal recessive	Opportunistic infection, failure to thrive, developmental delay	[25,26]
1b. CD4: CD8: 1–2					
CVID	Ratio 1–2 Overall (6 with ratio <1.0) Ref [27] N = 15 Ref [28] N = 9	III-IV	ICOS deficiency Autosomal recessive	Viral infections, opportunistic infection, increased cancer risk	[27,28]
CVID	Ratio 1–2 * N = 66	Unable to determine	CTLA4 Autosomal dominant	Hypogammaglobulinemia, respiratory infection, GI infection	[29]
1c. CD4/CD8 > 2					
Wiskott–Aldrich	Ratio > 20 N = 27	Unable to determine	WAS X-linked	Bleeding, autoimmunity, B-cell lymphoma	[30,31]

* Exact ratios are not reported; however, the authors note that lymphopenia affected CD8+ cells more than CD4+ cells.

**Table 3:** Immunodeficiencies with normal B cells and antibody.

Disease	CD4/CD8 Ratio	Estimated IHG Class	Genetic Defect and Inheritance	Associated Features	Reference
2a. CD4/CD8 < 1					
Mag T1 Deficiency	Ratio < 1 in 10/15 N = 15	III-IV	MAGT1 X-linked	Severe EBV, lymphoma, respiratory and GI infection	[32]
2b. CD4/CD8 1–2					
Ataxia–telangiectasia	Ratio 1–2 *	I-II	11q22-23 Autosomal recessive	Sinopulmonary infection, cerebellar ataxia, telangiectasias	[33]
2c. CD4/CD8 > 2	Ratio > 2 N = 32	Unable to determine	Zap70 Autosomal recessive	Autoimmunity	[34]

* Based on absolute CD4+ and CD8+ count medians. CD3+ CD4+ median 473 (range 99–1448) and CD3+ CD8+ median 350 (range 167–724).

## Data Availability

No data was created for this work, but if there is something of value in this work we will work to make it available.
